# Consumer Acceptance and Perceived Sensory Characteristics of Commercial Vegan Mayonnaise

**DOI:** 10.3390/foods14091542

**Published:** 2025-04-28

**Authors:** Juyoun Lee, Kyunghee Kim

**Affiliations:** Department of Food and Nutrition, Duksung Women’s University, Seoul 01369, Republic of Korea

**Keywords:** vegan mayonnaise, Check-All-That-Apply, sensory evaluation, plant-based products

## Abstract

This study aims to investigate the sensory characteristics of commercially available vegan mayonnaise using the Check-All-That-Apply (CATA) methodology and to determine the acceptability factors influencing consumer purchase intention. Six mayonnaise samples were evaluated by 112 consumers: one conventional mayonnaise and five commercially available vegan mayonnaises (labeled OGM, VVM, EBM, VM, SM, and OVM). Except for fatty flavor, rancid odor, artificial flavor, mouthcoating, melting, and mouthfeel, 15 characteristics (yellowness, glossiness, slimness, thickness, smoothness, beany odor, lemon aroma, nutty flavor, sourness, saltiness, sweetness, savory flavor, off-flavor, goes well with vegetables, and spreads well on crackers) were significantly different among 6 samples (*p* < 0.001). Across all evaluation attributes, OGM and VM had the highest acceptance, with no significant differences between the two samples except for overall taste. The VM was the only vegan mayonnaise that produced results similar to those of OGM, which is regular mayonnaise. The results of the study suggest that vegan mayonnaise can be a substitute for regular mayonnaise. We hope that this research will provide data that can be used as a basis for developing vegan mayonnaise products that meet the needs of consumers and food companies.

## 1. Introduction

Mayonnaise, consumed globally for its unique flavor and smooth texture, is commonly served with foods such as sandwiches, burgers, salads, and chicken, making it a staple in the food service industry [1]. Mayonnaise is a semi-solid oil-in-water (O/W) emulsion composed of oil, vinegar, egg yolk, and spices. While traditional mayonnaise typically contains 70–80% oil and egg yolks with high cholesterol levels, it remains the most widely used emulsifier due to its exceptional stability and high emulsifying power [2]. The global mayonnaise market was valued at USD 11.3 billion in 2022 and it is projected to grow from USD 11.8 billion in 2024 to USD 15.4 billion by 2030 [3]. In recent years, consumer demand for healthier foods has been on the rise, and this new trend has impacted mayonnaise formulations [4]. The global eggless mayonnaise market was valued at USD 4.1 billion in 2022 and is expected to grow at a CAGR of 6.2% from 2023 to 2032 [5]. This shift is driven by growing consumer awareness of health concerns, such as the high-calorie content of traditional mayonnaise and the risks associated with cholesterol and fatty acid oxidation in eggs [6,7,8]. Studies have demonstrated that plant-based diets, such as Mediterranean, vegetarian, and vegan diets, lower the risk of cardiovascular disease [9]. Particularly, vegan products utilizing beans are being developed as they offer plant proteins capable of substituting the value of animal proteins. Consumers are increasingly adopting plant-based diets (PBDs) due to their perceived health benefits and contributions to environmental sustainability. Consumer eating patterns significantly influence health, societal well-being, and the global environment [10,11]. Driven by demand for sustainable food that is ethical, eco-friendly, and health-conscious, the food industry is actively reformulating products and incorporating natural ingredients [1]. Creating an egg substitute is challenging from a food production perspective, not only from a nutritional standpoint, but also because of the many functions of eggs that affect the flavor, texture, and mouthfeel of mayonnaise [12]. In response to these trends, research has focused on producing mayonnaise using wheat protein, soy drink, peas, chickpeas, lentils, and aquafaba as substitutes for egg yolks [13,14,15,16]. Stability, viscosity, and color factors were similar in mayonnaise with egg yolks replaced by soy drink, and sensory evaluation showed no statistical difference up to the 50% replacement level [14]. The texture of the egg-free mayonnaise with chickpeas did not differ from the control, nor did the sensory attributes in the panel acceptance test [16]. Another study compared the physicochemical properties of traditional commercial mayonnaise and vegan dressing-type mayonnaise and found differences in physicochemical properties [17]. These results are due to the oils, thickeners, and various additives and processing conditions in mayonnaise. The structure, texture, and appearance of mayonnaise are not only important factors in determining consumer choice and satisfaction, but also its sensory properties [18]. Therefore, it is meaningful to investigate the factors that influence the sensory characteristics and acceptance of commercially available vegan mayonnaise among a panel of consumers. The food culture is changing around the world. Various consumption choices are available, and they change according to the values of personal health and social ethics. Vegetarianism is recommended for the environment, but it is not easy for everyone to follow a natural diet. However, a delicious and healthy sauce is one way to help you eat vegetarian. This study aims to investigate the sensory characteristics and acceptance of vegan mayonnaise currently available in the domestic market. It also aims to inform quality improvement of vegan mayonnaise by identifying sensory attributes that influence purchase intentions of a panel of consumers.

## 2. Materials and Method

### 2.1. General Characteristics

Table 1 presents the general demographic information about the survey respondents. The sample included 112 women, with the majority (68.8%) aged ≤20 years. Additionally, 79.5% of the participants were single. Regarding education level, 78.6% of respondents were university students or university graduates. Among the respondents, 76.8% indicated that they purchase vegan food, and 89.3% reported having consumed mayonnaise.

### 2.2. Materials

For this experiment, six commercially available mayonnaise products were selected (Table 2). Vegan mayonnaise was searched through Coupang and Naver, the most used shopping sites by Koreans in 2023. A total of 13 vegan mayonnaises were searched. There were 8 types of plain vegan mayonnaise without any other ingredients added. The researchers used the filters on the Coupang website and selected the five products with the highest sales volume for the study. Regular mayonnaise was also selected using the same method. Six types of mayonnaise were purchased through Coupang on 1 July 2023. To evaluate the differences in the organoleptic properties between vegan mayonnaise and regular mayonnaise, Ottogi Gold Mayonnaise (OGM; Ottogi, Eumseong, Republic of Korea), the product with the highest sales volume, was selected as the control group. The five vegan mayonnaise types tested were Eat’s Better Mayo (EBM; Wonil, Jincheon, Republic of Korea), ORGA Vege Mayo (OVM; Pulmuone, Seoul, Republic of Korea), Hello Veggie Plant-Based Soybean Mayo (SM; Ottogi, Eumseong, Republic of Korea), Vegan Mayo (VM; Kraft Heinz, Chicago, IL, USA), and VIVID Vegan Mayo (VVM; Dongwon, Asan-si, Republic of Korea). Table 3 and Table 4 show the nutritional information and ingredient list for each of them, reported in labeled brands.

### 2.3. Sample Preparation

Preliminary experiments were conducted with 10 panelists to identify and revise any unclear sections of the questionnaire. The preparation and presentation methods for the samples were finalized during this process. Each sample, weighing 12 g, was prepared on the morning of the test, placed in a transparent plastic container with a lid, refrigerated at 4 °C, and served within 15 min of removal from refrigeration. Each sample was assigned a unique three-digit random number, while the order of sample presentation was randomized using the Williams Latin Square Design [19] to minimize order-related biases. Participants were provided with six mayonnaise samples along with cucumber and carrot sticks (1 cm × 1 cm × 5 cm), IVY crackers (Haitia Confectionery, Jeonju-si, Jeollabuk-do, Republic of Korea), and bottled water (ICIS 8.0) (Lotte Chilsung, Cheongju-si, Chungcheongbuk-do, Republic of Korea) for evaluation.

### 2.4. Consumer Test 1: Check-All-That-Apply

The consumer panel included 112 female participants aged ≥19 years. Vegan diets are more often chosen by women than men, with 19% of men and 81% of women being vegan [20]. Studies on masculinity and vegetarianism have been conducted to promote a positive attitude toward vegetarianism, but while there has been a change in perception, there has been no change in attitude [21]. This is consistent with the findings that meat consumption is more suitable for men [22,23]. Therefore, this study chose to focus on women because it is evaluating vegan products. Participants were recruited online using Google Forms and the Duksung Women’s University website. This study was conducted in compliance with Institutional Review Board (IRB) procedures after receiving approval from the IRB of Duksung Women’s University (Approval No.: 2024-001-023-A). All participants voluntarily provided written informed consent before participating in the study. The descriptive terms used in the Check-All-That-Apply (CATA) survey were selected based on previous research on mayonnaise [24]. The final sensory characteristics included the following: Appearance—yellowness, glossiness, sliminess, thickness and smoothness; Aroma/Odor— beany odor, lemon aroma, rancid odor; Flavor/Taste—nutty flavor, fatty flavor, sourness, saltiness, sweetness, savory flavor, artificial flavor, off-flavor; and Texture—goes well with vegetables, spreadability on crackers, mouthcoating, melting and mouthfeel.

### 2.5. Consumer Test 2: Consumer Acceptance

The consumer panel included 112 women aged ≥19 who had no aversion to eating mayonnaise, were not allergic to soy, and had no dietary restrictions. The participants received a 30 min briefing on the research process, evaluation methods, rinsing protocols, evaluation criteria, and important precautions. The researchers clarified any questions that participants did not understand after reviewing the questionnaire to ensure accurate responses. Participants tasted mayonnaise samples in the order listed on the questionnaire and rated 12 attributes (appearance, color, overall taste, aftertaste, nutty flavor, flavor, viscosity, aroma, spreadability, harmony, oily, and overall acceptance). Each sample was rated on a 9-point structural hedonic scale. (1 = Dislike extremely, 5 = Neither like nor dislike, 9 = Like extremely). After evaluating all the samples, participants completed a questionnaire to provide demographic information, including gender, age, mayonnaise purchasing habits, frequency of mayonnaise consumption, foods typically eaten with mayonnaise, and awareness of vegan products.

### 2.6. Statistical Analysis

Chi-square tests and fisher’s exact test were performed to assess the significance between samples based on the frequency of selection data from the CATA responses for the sensory characteristics of the mayonnaise samples. Consumer acceptance and purchase intention for each sample were analyzed using analysis of variance (ANOVA) to determine significant differences between the samples. For traits exhibiting significant differences at the α = 0.05 level, Duncan’s multiple comparison test was performed. Additionally, partial least squares regression (PLSR) analysis was conducted to analyze the relationship between sensory characteristics by CATA and consumer acceptance. All statistical analyses were performed using XLSTAT software (version 2024.02, Addinsoft Inc., Paris, France).

## 3. Results and Discussion

### 3.1. Frequency of Sensory Attributes by CATA

Table 5 summarizes the sensory characteristics of mayonnaise as evaluated using 21 sensory attributes in the CATA method. Chi-square and Fisher’s exact test were conducted to identify characteristics that exhibited significant differences among the samples. Except for fatty flavor, rancid odor, artificial flavor, mouthcoating, melting, and mouthfeel, 15 characteristics (yellowness, glossiness, sliminess, thickness, smoothness, beany odor, lemon aroma, nutty flavor, sourness, saltiness, sweetness, savory flavor, off-flavor, goes well with vegetables, and spreads well on crackers) were significantly different among 6 samples (*p* < 0.001). The following sections provide a detailed breakdown of the characteristics identified by consumers in ≥50% of the samples. The OGM sample was characterized by attributes such as yellowness, glossiness, smoothness, nutty flavor, fatty flavor, sourness, goes well with vegetables, spreads well on crackers. The OVM sample exhibited thickness, nutty flavor, while the VVM sample was characterized by yellowness, sourness, spreads well on crackers. The EBM sample exhibited nutty flavor, sourness, the VM sample was defined by glossiness, smoothness, sourness, spreads well on crackers, and the SM sample was identified solely by sourness. Evaluation studies of food emulsifiers similar to mayonnaise using herbs and spice extracts can improve consumer acceptance and nutritional quality [25]. In terms of aroma/odor, it is a very meaningful result that no panelist felt a beany odor in either the OGM or the VM. The VM is the only vegan mayonnaise that does not contain soy drink or soybean powder isolate and contains spice powder. Adding spices seems to have a positive effect on the sensory test. In contrast, the four mayonnaise types with significantly lower consumer acceptance —OVM, VVM, EVM, and SM—were confirmed to contain soy drink or soy-based ingredients. Contrary to the previous studies, the study on the use of soy drink in mayonnaise demonstrated a reduction in cholesterol levels, improved storage stability, and organoleptic properties similar to those of regular mayonnaise [26,27]. Soybean protein isolate (SPI) is a high-quality protein additive characterized by its high protein content, which is achieved by removing most of the fat and carbohydrates. SPI exhibits several functional properties, such as emulsification, moisture retention, oil absorption, gelation, adhesion, and dispersion. Consequently, it is widely used in various food products, including meat, dairy, and flour-based items [28,29,30,31]. Table 4 shows that the OGM, OVM, EBM, and SM ingredients include soy protein isolate. This is consistent with the above research results, which were added for emulsification, gelling, adhesion, and dispersion. Izidoro, Dayane et al. [32] showed that in mayonnaise, fat affects flavor, mouthfeel, and texture characteristics. Sensory evaluation of two brands of commercial mayonnaise, two traditional mayonnaises and two low-fat mayonnaises showed that regular mayonnaise was rated more positively than low-fat mayonnaise, regardless of the brand. A similar result was found in a study by Karas et al. [33], in which traditional mayonnaise was rated better than the low-fat sample in terms of glossiness, homogeneity, mouthfeel, and overall acceptability. This shows that the fat content of mayonnaise is important. Karas et al. [33] found that traditional mayonnaise has a strong acidic odor, while low-fat mayonnaise has a less pronounced odor. It can be said that the sensory characteristics of acidity vary depending on the amount of fat. In OGM, OVM, EBM, VM, and SM, more than 50% of the panelists felt sourness, but only in OVM was it less than 50%. In this study, mayonnaise contained 80 g of OGM, 65 g of OVM, 52 g of VVM, 53 g of EBM, 71 g of VM, and 50 g of SM per 100 g (Table 3). The amounts of OGM and VM were similar, and the amounts of VVM, EBM, and SM were similar, with only OVM being different. The fat content may have affected the perception of sourness.

### 3.2. Evaluation of Consumer Acceptance

Table 6 presents the evaluation of consumer acceptance for the six mayonnaise samples. The evaluation of consumers was conducted based on the attributes of appearance, color, overall taste, aftertaste, nutty, flavor, viscosity, aroma, spreadability, harmony, oily, over acceptance using a 9-point hedonic scale. The test results showed significant differences between the six mayonnaise samples in all attributes (*p* < 0.001). Regarding color, no significant differences were observed between OGM, VM, and OVM. In all evaluated attributes, OGM and VM were the most preferred, and there was no significant difference between the two samples except for overall taste. The VM was the only vegan mayonnaise that produced results similar to those of OGM, which is regular mayonnaise. In the study by Izidoro, D. et al. [32], the fat content affected the flavor and texture of the sensory characteristics of mayonnaise. Karas, R. et al. [33] also found the same results, with regular mayonnaise being rated better than low-fat mayonnaise in terms of gloss, homogeneity, mouthfeel, and overall acceptability. The fact that OGM and VM have similar fat contents may have had a positive impact on consumer acceptance. The nutty was the most pronounced and highly rated by OGM and EBM. OVM was rated highly for color, aroma, and spreadability, while VVM was rated highly for viscosity, aroma, and spreadability. EBM was highly rated for its nutty and aroma. SM received the lowest rating in the attributes of appearance, color, overall taste, aftertaste, flavor, viscosity, harmony, oily, and overall acceptance. According to the research of Torrico, D.D. et al. [34], it can be seen that the amount of oil increases the saltiness and viscosity. In this study, Viscosity showed the best results for OGM, VVM, and VM, and the highest oil concentration for OGM and VM, which is consistent with the results. OVM, VVM, and EBM exhibited similar patterns in the hedonic scale, with nutty flavor and aroma being the most significant attributes.

### 3.3. Correlation Between Sensory Characteristics and Mayonnaise Acceptance

**Figure 1:**
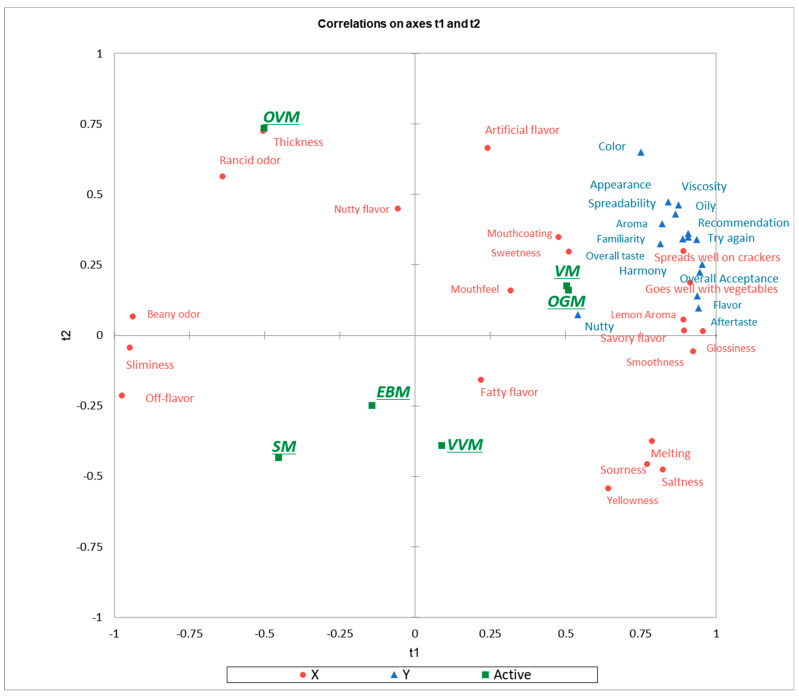
Correlation between the sensory characteristics and overall mayonnaise acceptance.

Figure 1 illustrates the correlation between the sensory characteristics of mayonnaise and consumer acceptance analyzed using PLSR. The taste factors and sensory attributes that were highly correlated with overall acceptance, excluding overall acceptance itself, included spreadability on crackers, goes well with vegetables, lemon aroma, glossiness, and savory flavor. The OGM and VM samples were most associated with these attributes. In contrast, the attributes negatively correlated with overall acceptance included rancid odor, thickness, beany odor, sliminess, and off-flavor. The OVM samples were most related to these negative attributes. Soybeans serve as a nutritious alternative to animal-based foods due to their content of unsaturated fatty acids, B vitamins, dietary fiber, iron, calcium, zinc, and other physiologically active compounds [30]. Given that soy protein is particularly popular among vegetarians, the food industry has expanded its product offerings by incorporating soybeans [35]. However, as most of the negative sensory characteristics come from soybeans, managing these undesirable traits is crucial. Mayonnaise is a sauce that is eaten with other foods rather than eaten on its own. It is eaten with sandwiches or salads and as a dipping sauce for vegetable sticks, so it is important that it goes well with other foods. Previous research has shown that the addition of vegetables, spices, and other ingredients reduces the inherent flavor of soybeans, improving sensory evaluation results [25]. Further research is necessary to develop vegan mayonnaise incorporating various vegetables and spices, enhancing its compatibility with diverse foods. With restaurants and cafes adopting plant-based menus, home sales accounting for the largest share of sales, and consumer preference for sustainable and allergy-free foods, vegan mayonnaise must be developed to meet consumer acceptance.

### 3.4. Assessment of Consumer Purchase Intentions for Mayonnaise

Table 7 shows the results of the consumer panel’s evaluation of their intention to purchase commercial vegan mayonnaise. The purchase intention of consumers was investigated using attributes such as try again, recommendation, and familiarity. All attributes were significantly different among samples (*p* < 0.001). In all three categories, OGMs received the highest ratings. Regarding the willingness to repurchase and intention to recommend, OGM and VM samples had the highest ratings, each scoring >6 points (*p* < 0.001). After OGM and VM, EBM demonstrated the next highest purchase intention. However, a significant difference in familiarity was observed between OGM and VM, potentially due to OGM being the most familiar mayonnaise brand among Koreans. Kerslake, E. and Collier, E. S. [36,37] found that familiarity is very important when choosing food. Participants may have chosen the OGM mayonnaise because of its familiar taste. However, the high purchase intention for VM indicates the positive potential of vegan products. As interest in vegetarianism grows, the market continues to expand while product offerings are diversifying to meet consumer demand. Previous studies identified flavor, texture, and taste as negative factors, along with concerns about new foods, high levels of food additives, and high prices [37,38,39]. The global shift to plant-based diets and the increasing prevalence of lactose intolerance and egg allergies have boosted the market for vegan mayonnaise. However, the main limitation of vegan mayonnaise is its high production cost, which makes it expensive. The ingredients used to make it, such as aquafaba, pea protein, and vegetable oils, are expensive, and the complex manufacturing process is required to achieve the desired taste, texture, and consistency. Therefore, reducing costs can be an important factor in expanding the market. Pouches offer portability, portion control, and can reduce the amount of material used. Pouch packaging is growing due to increasing consumer demand for eco-friendly and convenient packaging solutions, especially among younger and sustainability-focused demographics. According to Cognitive Market Research, plastic containers for vegan mayonnaise are expected to be used for mass distribution and storage convenience because they are lightweight and durable [40]. Consumer demand for healthier and environmentally friendly products must be addressed in commercial offerings.

## 4. Conclusions

Five commercially available vegan mayonnaise and one regular mayonnaise were selected to investigate consumer perception of sensory attributes identified using CATA and consumer acceptance. A total of 15 of the 21 sensory attributes of CATA were significantly different between samples: yellowness, glossiness, and sliminess, thickness, smoothness, and beany odor, lemon aroma, nutty flavor, sourness, and saltiness, sweetness, savory flavor, off-flavor and goes well with vegetables, spreads well on crackers (*p* < 0.001). Consumer acceptance tests showed that all 12 attributes were significantly different between the six mayonnaise samples (*p* < 0.001). The OGM and VM samples had the highest consumer acceptance. VM had the highest acceptance ratings for appearance, color, overall taste, aftertaste, flavor, viscosity, aroma and spreadability, harmony, oily, and overall acceptance. OGM had the highest acceptance for appearance, color, overall taste, aftertaste, nutty, flavor, viscosity, aroma and spreadability, harmony, and overall acceptance. Regular mayonnaise (OGM) and vegan mayonnaise (VM) had equally high acceptance across 9 of the 12 consumer acceptance attributes. A PLSR analysis of the correlation between sensory attributes of mayonnaise and consumer acceptance showed that spreadability on crackers, goes well with vegetables, lemon aroma, glossiness, and savory flavor were highly correlated with overall mayonnaise acceptance. Consumer interest in plant-based foods is growing and product development is underway, but often falls short of consumers’ taste needs. Vegan mayonnaise should be researched because it is healthy and necessary for consumers with dietary restrictions. Therefore, the fact that vegan mayonnaise and regular mayonnaise in this study showed fairly equally good sensory attributes and consumer acceptance is a positive result for consumers who are looking for diversity in their consumption. According to the results of this study suggest that vegan mayonnaise can be a substitute for regular mayonnaise. However, the study’s limitations include the fact that it was conducted in Korea, which makes it difficult to apply globally, and that it only evaluated a female panel. In future research, it would be helpful to study a variety of male and age-specific panels and to study the ingredients that affect the sensory evaluation of vegan mayonnaise. We hope that this research will provide data that can be used as a basis for developing vegan mayonnaise products that meet the needs of consumers and food companies.

## Figures and Tables

**Table 1:** General characteristics of survey respondents (*n* = 112).

Variables	*n*	%
Age group (years)		
19–29	77	68.8
30–39	19	17.0
≥40 years	16	14.3
Marital status		
Single	89	79.5
Married	23	20.5
Education level		
≤College graduate	6	5.4
University	88	78.6
≥Graduate school	18	16.1
Purchasing vegan food		
Yes	86	76.8
No	26	23.2
Experience consuming mayonnaise		
Yes	100	89.3
No	12	10.7

**Table 2:** List of information on mayonnaise samples.

Sample	Label	Company	Origin	Expiration Date	Amount (mL)
Ottogi Gold Mayonnaise	OGM	Ottogi Corp.	Republic of Korea	26 February 2024	200
ORGA Vege Mayo	OVM	Pulmuone Co., Ltd.	Republic of Korea	23 October 2023	300
VIVID Vegan Mayo	VVM	Dongwon Home Food Inc.	Republic of Korea	11 February 2024	250
Eat’s Better Mayo	EBM	The PlantEat Inc.	Republic of Korea	1 December 2023	245
Vegan Mayo	VM	The Kraft Heinz Company	USA	3 November 2023	270
Soybean Mayo	SM	Ottogi Corp.	Republic of Korea	4 January 2024	310

**Table 3:** Nutritional information of mayonnaise samples.

Nitritional Fact (100 g)	OGM		OVM		VVM		EBM		VM		SM	
Energy (Kcal)	730		602		520		525		680		500	
Sodium (mg)	510	26% ^(1)^	750	38%	550	28%	460	23%	750	38%	550	28%
Carbohydrate (g)	1	0%	2	0.6%	12	4%	12	4%	10	3%	11	3%
Sugar(g)	1	1%	2.5	2.3%	2.5	3%	2	2%	6	6%	5	5%
Fat (g)	80	148%	65	121%	52	96%	53	98%	71	131%	50	93%
Trans-fatty acids (g)	<0.5		0.2		0.9		<0.5		0		0.5	
Saturated fat (g)	14	93%	6	38%	10	67%	8	53%	4.9	33%	9	60%
Cholesterol (mg)	55	18%	0	0%	0	0%	0	0%	0	0%	0	0%
Protein (g)	1	2%	1.3	2.3%	1	2%	1	2%	0.1	0%	1	2%

^(1)^ % Daily Value; Percent Daily Values are based on a 2000 calorie diet.

**Table 4:** Ingredient list of mayonnaise samples ^(1)^.

Ingredient	OGM	OVM	VVM	EBM	VM	SM
Vegetable oil	O	-	-	-	-	O
Canola oil	-	O	-	-	O	-
Soybean oil	-	-	O	O	-	-
Purified water	O	O	O	O	O	O
Sugar	O	O	O	O	-	O
Refined salt	O	O	O	O	O	O
Fermented vinegar	O	O	O	O	O	O
Soybean protein isolate	O	O	-	O	-	O
Lemon concentrate	-	O	O	O	O	-
Xanthan gum	O	O	O	O	-	O
Modified starch	-	O	O	-	-	O
Antioxidant	O	-	O	-	O	-
Lactic acid	-	-	O	-	-	O
Yeast extract	-	-	O	-	-	O
Fructose	-	O	-	-	O	-
Egg yolk liquid	O	-	-	-	-	-
Egg white liquid	O	-	-	-	-	-
Flavoring oil	O	-	-	-	-	-
Plant-derived protein	O	-	-	-	-	-
Enzyme	O	-	-	-	-	-
Soy sauce mix	O	-	-	-	-	-
Guar gum	-	O	-	-	-	-
Dextrin	-	O	-	-	-	-
Almond paste	-	O	-	-	-	-
Compound seasoning	-	O	-	-	-	-
Sodium 5′-ribonucleotide	-	O	-	-	-	-
Soy drink	-	-	O	-	-	-
Emulsifier	-	-	O	-	-	-
Gardenia yellow	-	-	O	-	-	-
Glycerin fatty acid ester	-	-	-	O	-	-
D-tocopherol	-	-	-	O	-	-
Natural flavoring	-	-	-	O	-	-
Steamed soybean- powder	-	-	-	O	-	-
Black soybean-powder	-	-	-	O	-	-
Maize Thickener	-	-	-	-	O	-
Onion powder	-	-	-	-	O	-
Natural color(160a)	-	-	-	-	O	-
Mustard powder	-	-	-	-	-	O
Nutritional fortifier	-	-	-	-	-	O
Natural flavoring	-	-	-	-	-	O
Tamarind gum	-	-	-	-	-	O

^(1)^ Source: Information described on the label of each product.

**Table 5:** Sensory characteristics evaluation results based on the Check-All-That-Apply method.

		OGM		OVM		VVM		EBM		VM		SM		*p*-Value ^(1)^
		*n*	%	*n*	%	*n*	%	*n*	%	*n*	%	*n*	%	
Appearance	Yellowness	66	58.41	30	26.55	81	71.68	50	44.25	54	47.79	45	39.82	<0.001
	Glossiness	101	89.38	22	19.47	51	45.13	48	42.48	102	90.27	40	35.40	<0.001
	Sliminess	9	7.96	23	20.35	11	9.73	19	16.81	9	7.96	27	23.89	<0.001
	Thickness	25	22.12	72	63.72	34	30.09	15	13.27	29	25.66	31	27.43	<0.001
	Smoothness	79	69.91	24	21.24	49	43.36	54	47.79	61	53.98	26	23.01	<0.001
Aroma/odor	Beany odor	-	-	46	40.71	20	17.70	39	34.51	-	-	31	27.43	<0.001
	Lemon aroma	20	17.70	7	6.19	19	16.81	13	11.50	35	30.97	6	5.31	<0.001
	Rancid odor	8	7.08	14	12.39	5	4.42	8	7.08	9	7.96	12	10.62	0.307
Flavor/ Taste	Nutty flavor	63	55.75	64	56.64	42	37.17	73	64.60	53	46.90	47	41.59	<0.001
	Fatty flavor	57	50.44	41	36.28	53	46.90	31	27.43	36	31.86	48	42.48	0.002
	Sourness	67	59.29	38	33.63	59	52.21	57	50.44	74	65.49	62	54.87	<0.001
	Saltiness	37	32.74	14	12.39	37	32.74	24	21.24	36	31.86	28	24.78	<0.001
	Sweetness	11	9.73	15	13.27	14	12.39	17	15.04	33	29.20	7	6.19	<0.001
	Savory flavor	34	30.09	12	10.62	17	15.04	23	20.35	35	30.97	18	15.93	<0.001
	Artificial flavor	12	10.62	14	12.39	12	10.62	5	4.42	12	10.62	8	7.08	0.323
	Off-flavor	4	3.54	35	30.97	23	20.35	31	27.43	7	6.19	42	37.17	<0.001
Texture	Goes well with vegetables	59	52.21	32	28.32	38	33.63	46	40.71	55	48.67	25	22.12	<0.001
	Spreads well on crackers	83	73.45	54	47.79	72	63.72	44	38.94	82	72.57	34	30.09	<0.001
	Mouthcoating	54	47.79	37	32.74	34	30.09	27	23.89	34	30.09	34	30.09	0.005
	Melting	41	36.28	34	30.09	41	36.28	40	35.40	39	34.51	35	30.97	0.867
	Mouthfeel	25	22.12	27	23.89	26	23.01	26	23.01	35	30.97	28	24.78	0.671

^(1)^ *p*-value obtained through chi-square test and fisher’s exact test.

**Table 6:** Evaluation of consumer acceptance for six mayonnaise varieties ^(1)^.

	OGM		OVM		VVM		EBM		VM		SM		*p*-Value ^(2)^
	Mean	SD	Mean	SD	Mean	SD	Mean	SD	Mean	SD	Mean	SD	
Appearance	7.60 ^a^	1.24	5.78 ^b^	1.73	5.97 ^b^	1.55	4.99 ^bc^	1.87	7.52 ^a^	1.38	4.23 ^c^	2.11	<0.001
Color	7.56 ^a^	1.24	6.51 ^ab^	1.76	5.84 ^b^	1.61	5.08 ^bc^	1.91	7.60 ^a^	1.31	4.27 ^c^	2.02	<0.001
Overall taste	6.48 ^a^	1.88	4.93 ^c^	2.03	5.26 ^bc^	1.79	5.46 ^bc^	2.00	6.29 ^b^	1.84	4.60 ^c^	2.10	<0.001
Aftertaste	6.19 ^a^	1.84	4.77 ^c^	1.90	5.18 ^bc^	1.93	5.53 ^b^	2.05	6.15 ^a^	2.06	4.73 ^c^	2.02	<0.001
Nutty	6.25 ^a^	1.79	5.35 ^b^	1.90	5.33 ^b^	1.81	6.34 ^a^	2.09	5.74 ^ab^	1.99	5.10 ^b^	2.18	<0.001
Flavor	6.42 ^a^	1.75	4.93 ^c^	2.07	5.31 ^b^	1.75	5.65 ^b^	1.90	6.22 ^a^	1.94	4.77 ^c^	2.11	<0.001
Viscosity	7.18 ^a^	1.24	5.73 ^b^	1.96	6.19 ^ab^	1.49	5.08 ^bc^	1.98	6.99 ^a^	1.30	4.22 ^c^	2.08	<0.001
Aroma	5.92 ^a^	1.85	5.27 ^a^	1.80	5.33 ^a^	1.80	5.53 ^a^	1.82	5.61 ^a^	1.86	4.83 ^b^	1.90	<0.001
Spread ability	7.33 ^a^	1.36	6.27 ^ab^	1.95	7.02 ^a^	1.52	5.45 ^b^	2.27	7.52 ^a^	1.34	4.63 ^c^	2.32	<0.001
Harmony	6.52 ^a^	1.75	5.27 ^bc^	1.87	5.75 ^b^	1.91	5.62 ^b^	2.01	6.67 ^a^	1.77	4.70 ^c^	2.08	<0.001
Oily	6.10 ^ab^	1.79	5.59 ^b^	1.94	5.68 ^b^	1.79	5.42 ^b^	1.92	6.37 ^a^	1.72	4.70 ^c^	2.11	<0.001
Overall acceptance	6.48 ^a^	1.86	4.99 ^bc^	2.12	5.22 ^b^	1.82	5.13 ^bc^	1.93	6.49 ^a^	1.83	4.35 ^c^	2.16	<0.001

^(1)^ 9-point hedonic scale: 1 = Dislike extremely, 5 = Neither like nor dislike, 9 = Like extremely; ^(2)^ *p*-value obtained through one-way analysis of variance; ^a–c^ Indicates significant differences between the samples for each consumer acceptance.

**Table 7:** Evaluation of consumer purchase intentions for six mayonnaise varieties.

Mayonnaise Sample	OGM		OVM		VVM		EBM		VM		SM		*p*-Value ^(1)^
	Mean	SD	Mean	SD	Mean	SD	Mean	SD	Mean	SD	Mean	SD	
Try again	6.59 ^a^	2.10	4.73 ^c^	2.40	4.81 ^c^	2.38	5.16 ^b^	2.39	6.42 ^a^	2.11	3.91 ^d^	2.42	<0.001
Recommendation	6.63 ^a^	2.08	4.67 ^b^	2.29	4.71 ^b^	2.22	4.97 ^b^	2.34	6.34 ^a^	2.05	3.88 ^c^	2.40	<0.001
Familiarity	7.42 ^a^	1.93	4.39 ^c^	2.21	4.74 ^c^	2.22	4.09 ^cd^	2.25	6.14 ^b^	2.09	3.50 ^d^	2.26	<0.001

^(1)^ *p*-value from one-way analysis of variance; ^a–d^ Indicates significant differences between samples for each purchase intention category.

## Data Availability

The original contributions presented in the study are included in the article, further inquiries can be directed to the corresponding author.
